# A modified method for genomic DNA extraction from the fish intestinal microflora

**DOI:** 10.1186/s13568-018-0578-3

**Published:** 2018-04-02

**Authors:** Zhuoran Han, Jingfeng Sun, Aijun Lv, YeongYik Sung, Xueliang Sun, Hongyue Shi, Xiucai Hu, Anli Wang, Kezhi Xing

**Affiliations:** 1 Tianjin Key Lab of Aqua-ecology and Aquaculture, Fisheries College, Tianjin Agricultural University, Tianjin, 300384 China; 2 Key Laboratory of Ecology and Environment Science of Higher Education Institutes, Guangdong Provincial Key Laboratory for Healthy and Safe Aquaculture, College of Life Science, South China Normal University, Guangzhou, 510631 China; 3 Department of Aquaculture Science, Faculty of Fisheries and Aqua-Industry, University Malaysia Terengganu, 21030 Kuala Terengganu, Malaysia

**Keywords:** Modified method, Genomic DNA extraction, Fish intestinal microflora, OD_260_/OD_280_ ratio, DNA concentration

## Abstract

A modified genomic DNA extraction method named the combination of lysozyme and ultrasonic lysis (CLU) method was used to analyze the fish intestinal microflora. In this method, the physical disruption and chemical lysis steps were combined, and some parameters in the key steps were adjusted. In addition, the results obtained by this method were compared with the results obtained by the Zirmil-beating cell disruption method and the QIAamp Fast DNA Stool Mini Kit. The OD_260_/OD_280_ ratio and concentration of the DNA extracted using the CLU method were 2.02 and 282.8 µg/µL, respectively; when the incubation temperatures for lysozyme and RNase were adjusted to 37 °C, those values were 2.08 and 309.8 µg/µL, respectively. On the agarose gel, a major high-intensity, discrete band of more than 10 kb was found for the CLU method. However, the smearing intensity of degraded DNA was lower when the incubation temperatures were 60 °C for lysozyme and 30 °C for RNase than when incubation temperatures of 37 °C for lysozyme and 37 °C for RNase were used. The V3 variable region of the prokaryotic 16S rDNA was amplified, and an approximately 600-bp fragment was observed when the DNA extracted using the CLU method was used as a template. The CLU method is simple and cost effective, and it yields high-quality, unsheared, high-molecular-weight DNA, which is comparable to that obtained with a commercially available kit. The extracted DNA has potential for applications in critical molecular biology techniques.

## Introduction

The gut microbiota is associated with many key functions of the host, such as resistance to infectious diseases and the decomposition of nutrients, and it provides the host with physiologically active materials, such as enzymes, amino acids and vitamins (Sugita et al. 1997). An altered microbiota in the intestine can lead to altered host immune function, as well as an increased risk of disease (Brown et al. 2012; Morgan et al. 2012). For fish, the gut microbiota also plays important roles in health and physiology (Ganguly and Prasad 2011). Over the last decade, investigations of the intestinal microbiota of fish have aimed to study its significant biological functions and make use of probiotic bacteria (Gatesoupe 1999; Narrowe et al. 2015; Xia et al. 2014). In addition, these studies were expected to contribute in a meaningful way to enhancing immunity and reducing mortality in cultured fish.

In early studies, conventional culture-dependent techniques were used; however, only a small percentage of the bacterial flora was identified (Kathiravan et al. 2015). Recently, molecular techniques, including but not limited to denatured gradient gel electrophoresis (Li et al. 2011), fluorescence in situ hybridization (Hoffmann et al. 2006) and 16S rDNA high-throughput sequencing (Gajardo et al. 2016), have been used successfully to analyze the complex microbial community from fish intestines. Studies using molecular techniques have retrieved many novel sequences, which could not be identified as part of the intestinal flora of fish with traditional culture-dependent methods.

DNA extraction from the intestinal microflora is the key step for molecular biological analysis. Several protocols for extracting DNA from the fish intestinal microflora, including physical and chemical methods, have been described. Generally, common physical disruption methods have been employed, such as freezing–thawing (Fan et al. 2014), sonication (Yang et al. 2006) and bead beating (Carrigg et al. 2008). In addition, a variety of chemical lysis approaches, including cetyl trimethyl ammonium bromide (Chapela et al. 2007) and Triton X-100 (Wang et al. 2012), have been used to obtain higher purity DNA. The enzymatic digestion method for DNA extraction frequently employs lysozyme and proteinase K to quicken the process and increase the DNA yield. In recent years, commercial kits have also been chosen to extract genomic DNA from the gut microbiota because of their high efficiency, simple operation, and lack of wasted time. There are many kinds of kits for extracting microbial genomic DNA, such as the QIAamp Fast DNA Stool Mini Kit (Qiagen, Germany) (Xia et al. 2014), the Power Soil DNA Isolation Kit (Mo Bio, USA) (Stearns et al. 2011) and the Minibest Bacterial Genomic DNA Extraction Kit (Takara, Japan) (Hu et al. 2013). Unfortunately, most of these kits were designed for samples from mammals, the soil environment or a single type of microbe. In addition, the occurrence of degradation of the DNA extracted from the fish intestinal microflora is not avoidable with these kits and usually results in divergent analysis results in relation to the diversity of the microbial community.

To acquire purified and minimally degraded DNA from the fish intestinal microflora for further molecular biological study and subsequent analysis of microbial communities, we attempted to obtain an improved method. In this method, the physical disruption and chemical lysis steps were combined, and some parameters in the key steps were adjusted. For convenience, the name for the method was assigned as the combination of lysozyme and ultrasonic lysis (CLU) method. Herein, we introduce this method and its application for DNA extraction from the intestinal microflora of the koi carp *Cyprinus carpio* var. *Koi*. The results were compared with those obtained by the Zirmil-beating cell disruption (ZBC) method, referring to the research of Zoetendal et al. (2006), and the QIAamp Fast DNA Stool Mini Kit (QIA, Qiagen, Hilden, Germany), a common commercial kit. In addition, two other species of fish, the half-smooth tongue sole *Cynoglossus semilaevis* and the Jian carp *Cyprinus carpio* var. *Jian*, were used for intestinal microflora genomic DNA extraction using the CLU method.

## Materials and methods

### Fish

The *C. carpio* var. *Koi* (380–410 g) and the *C. carpio* var. *Jian* (18–20 g) were provided by the Gongwang Fish Breeding Center in Tianjin. *C. semilaevis* (130–145 g) specimens were provided by the Tianjin Haifa Aquaculture Center. The fish were transferred back to Tianjin Agricultural University and maintained under optimal rearing conditions (*C. carpio* var. *Koi* and *C. carpio* var. *Jian*: 20 °C, pH 7.5; *C. semilaevis*: 23 °C, pH 7.5, salinity 22 ppt) for 1 week. Aeration was provided to maintain optimal dissolved oxygen, and the fish were fed with commercial formulated pellets twice daily.

### Sample preparation

Prior to dissection for sample collection, all fish were euthanized with an overdose of MS-222 (Sigma-Aldrich, St Louis, MO, USA). The exterior of the fish was wiped clean with 70% ethanol, the abdomen was opened at the ventral midline and the whole intestine was aseptically removed from the abdominal cavity. All experimental procedures on the fish were approved by the Animal Care Committee of Tianjin Agricultural University. The methods were performed in accordance with the approved guidelines and regulations.

The gut samples were used directly after their removal from the fish. The intestinal contents and mucosa of three individuals were collected, pooled together and homogenized mechanically by pestle three times for 1 min each using a hand-held homogenizer. The sample was centrifuged at 110*g* for 5 min at 4 °C, and the supernatant was placed into a new, sterile 50-mL centrifuge tube. Subsequently, the supernatant was centrifuged at 2700*g* for 5 min. The precipitated bacteria were resuspended with 4 mL of sterile phosphate-buffered saline (PBS; 10 mM, pH 7.2; Dingguo Changsheng, Beijing, China), and the suspension was divided into quadruplicate samples with a volume of 1 mL. Four subsamples with the same volume and concentration from *C. carpio* var. *Koi* were used for the extraction of genomic DNA with the CLU (lysozyme/RNase: 37 °C/37 °C), CLU (lysozyme/RNase: 60 °C/30 °C), ZBC, and QIA methods.

Subsequently, to confirm the applicability of the CLU method for other fish species, *C. semilaevis* and *C. carpio* var. *Jian* were also used for the extraction of intestinal microflora genomic DNA using the CLU (lysozyme/RNase: 60 °C/30 °C) method.

### DNA extraction

#### CLU method

A bacteria suspension (1 mL) was dispensed into a 2-mL microtube and disrupted 50 times for 2 s each with the Ultrasonic Cell Disruption System (Ningbo Scientz Biotechnology, Ningbo, China), with an interval of 5 s. After centrifugation at 21,500*g* at 4 °C for 5 min, the upper aqueous layer was discarded. The sample was disrupted by incubation at 60 °C for 30 min after adding 750 µL of TE buffer (10 mM Tris-HCl, 1 mM EDTA, pH 8.0) and 50 µL of lysozyme (20 mg/mL; Sangon Biotech, Shanghai, China). Subsequently, 10 µL of RNase A (20 µg/mL; Sangon Biotech) was added to the centrifuge tube, and the suspension was then incubated at 30 °C for 30 min. The tube was then incubated at 65 °C for 60 min with inversion every 20 min after adding 100 µL of 10% SDS (pH 7.4; Sigma-Aldrich) and 30 µL of proteinase K (20 mg/mL; Sangon Biotech). Thereafter, an equal volume of phenol:chloroform:isoamyl alcohol (25:24:1) was added, and the sample was mixed by inversion. The sample was centrifuged at 21,500*g* for 2 min, and the supernatant was collected in a new 2-mL sterile centrifuge tube. An equal volume of chloroform: isoamyl alcohol (24:1) was added to the tube, and the suspension was mixed gently and centrifuged at 21,500*g* for 2 min. The upper aqueous layer was transferred to another 2-mL sterile centrifuge tube, and the DNA was precipitated using a 1/10 volume of NaAc (3 M, pH 5.2) and 2 volumes of ice-cold (− 20 °C) 95% ethanol, followed by centrifugation at 21,500*g* for 5 min at 4 °C. The DNA pellet was washed twice using 1 mL of 70% ethanol before being air dried and finally resuspended in 100 µL of TE buffer (preheated to 50 °C).

This protocol was also used for samples from *C. carpio* var. *Koi*, *C. semilaevis*, and *C. carpio* var. *Jian*. In this study, when the CLU method is mentioned in the text, incubation temperatures of 60 °C for lysozyme and 30 °C for RNase were used, unless specified otherwise. To investigate the influence of the incubation temperatures of lysozyme or RNase on the DNA extracted in the CLU method, 37 °C was also used for the incubation temperatures of lysozyme and RNase during the process of extracting genomic DNA from the intestinal microflora of *C. carpio* var. *Koi*.

#### ZBC method

DNA was extracted from 1 mL of a bacterial suspension according to the method of Zoetendal et al. (2006). The bacteria suspension was transferred to a 2-mL Lysing Matrix A tube (MP Biomedicals, Santa Ana, USA), followed by the addition of 150 µL of buffer-saturated phenol to the tube. The sample was oscillated by 4 m/s for 2 min with the FastPrep^®^-24 Instrument (MP Biomedicals), and cooled on ice for every 30 s and purified with 150 µL of chloroform: isoamyl alcohol (24:1), and then centrifuged at 21,500*g* at 4 °C for 2 min. Thereafter, an equal volume of phenol:chloroform:isoamyl alcohol (25:24:1) was added, and the sample was mixed by inversion. The sample was centrifuged at 21,500*g* for 2 min, and the supernatant was transferred to a new 2-mL sterile centrifuge tube. This step was repeated until the interface of the two layers was clean. An equal volume of chloroform: isoamyl alcohol (24:1) was added to the tube, and the sample was mixed gently and centrifuged at 21,500*g* for 2 min; the supernatant was then transferred into another new 2-mL centrifuge tube. DNA was precipitated with a 1/10 volume of 3 M NaAc (pH 5.2) and 2 volumes of cold 95% ethanol (− 20 °C) and stored at − 20 °C for 30 min. Thereafter, the samples were centrifuged at 21,500*g* for 20 min, and the supernatant was discarded. The DNA was washed with 500 µL of cold (− 20 °C) 70% ethanol and centrifuged at 21,500*g* for 5 min at 4 °C. The DNA pellet was dried by placing the tube upside down on tissue paper for 15 min, and the dried DNA was rehydrated in 100 µL of TE buffer (pH 8.0).

#### QIA method

One milliliter of the bacterial suspension was centrifuged at 2700*g* for 5 min, and the precipitated bacteria were resuspended with 220 µL of PBS. DNA was extracted from 220 µL of the bacterial suspension using a QIAamp Fast DNA Stool Mini Kit (Qiagen) according to the manufacturer’s instructions.

### Evaluation of the quality of the extracted DNA

To evaluate the extracted DNA, all types of products were treated 4 times. The quality of the extracted DNA was evaluated according to the absorbance ratios at 260/280 nm. The quantity of the extracted DNA was evaluated using the absorbance at 260 nm, which was converted into ng/μL of double-stranded DNA using the established conversion factor of 50 ng/μL for one optical density unit at 260 nm (Sambrook et al. 1989). Agarose gel electrophoresis of the isolated DNA samples extracted with all three methods was carried out in 0.8% gels, and the gels were observed under a Gel Doc System (Thermo Fisher Scientific, Waltham, MA, USA).

### PCR amplification

Amplification of variable region V3–V4 of the 16S rDNA was performed using the genomic DNA extracted with different methods as templates. PCR was conducted using the universal primers 341F (5′-CCCTACACGACGCTCTTCCGATCTGCCTACGGGNGGCWGCAG-3′) and 805R (5′-GACTGGAGTTCCTTGGCACCCGAGAATTCCAGACTACHVGGGTATCTAATCC-3′) (Li et al. 2016). Each reaction was performed in a 50-μL volume containing 20 ng of bacterial DNA, 5 µL of 10× PCR buffer, 0.5 µL of dNTP (10 mM), 0.5 µL of PCR primer F (50 µM), 0.5 µL of Primer R (50 µM) and 0.5 µL of Platinum Taq (5 U/µL). The samples were amplified using a T100™ Thermal Cycler (BioRad Laboratories, Hercules, CA, USA) with the following conditions: initial denaturation at 94 °C for 3 min; 5 cycles of 94 °C for 30 s, 45 °C for 20 s, and 65 °C for 30 s; and 20 cycles of 90 °C for 30 s, 55 °C for 20 s, and 72 °C for 30 s, followed by a final elongation at 72 °C for 5 min. The PCR products were separated by electrophoresis in 2% agarose gels.

## Results

### Quality of extracted DNA

Both the quality and quantity of the extracted DNA were assessed by measuring the absorbance at wavelengths of 260 nm and 280 nm (Table 1) and by visualizing the extracted community DNA on agarose gels. The OD_260_/OD_280_ ratio and the concentration of the extracted DNA for the CLU method were 2.02 and 282.8 µg/µL, respectively. When the incubation temperatures for lysozyme and RNase were adjusted to 37 °C, those values were 2.08 and 309.8 µg/µL, respectively. The OD_260_/OD_280_ ratios of the DNA extracted using the ZBC and QIA methods were 2.07 and 2.09, respectively. In addition, the DNA concentrations of those samples were 1002.6 µg/µL and 161.6 µg/µL, respectively.

**Table 1:** Purity and concentration of the extracted genomic DNA

Method	IT (lysozyme/RNase)	DNA (ng/µL)	DNA (ng/µL) range	A260/280	A260/280 range	DNA integrity
CLU	37 °C/37 °C	309.80 ± 66.79	133–448	2.083 ± 0.141	1.819–2.423	++
	60 °C/30 °C	282.80 ± 85.09	50–478	2.020 ± 0.161	1.701–2.407	++
ZBC	NA	1002.60 ± 365.15	300–2352	2.074 ± 0.147	1.712–2.413	+
QIA	NA	161.60 ± 54.35	82.5–373	2.089 ± 0.096	1.810–2.224	++

Values are represented as the mean ± SE; DNA integrity was assayed by agarose electrophoresis and indicated as good (++) or sufficient (+) based on the band clarity, density and presence of smearing

*NA* not available, *IT* incubation temperature

**Fig. 1:**
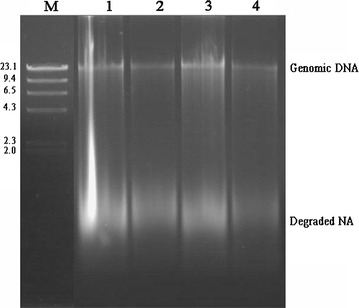
Agarose gel electrophoresis of the genomic DNA extracted from the intestinal microflora of *Cyprinus carpio* var. *Koi* using the CLU method. Lanes: *M*, molecular marker. 1, incubation temperatures of 37 °C for lysozyme and 37 °C for RNase were used; 2, incubation temperatures of 37 °C for lysozyme and 30 °C for RNase were used; 3, incubation temperatures of 60 °C for lysozyme and 37 °C for RNase were used; and 4, incubation temperatures of 60 °C for lysozyme and 30 °C for RNase were used

**Fig. 2:**
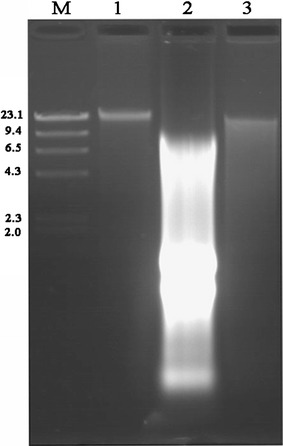
Agarose gel electrophoresis of the genomic DNA extracted from the intestinal microflora of *Cyprinus carpio* var. *Koi*. Lanes: *M* molecular marker. 1, CLU method; 2, ZBC method; and 3, QIA method

**Fig. 3:**
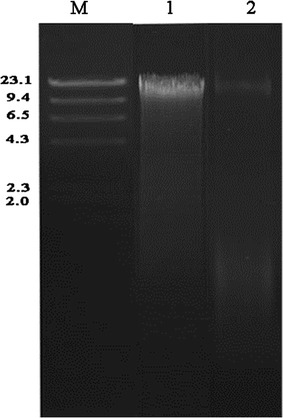
Agarose gel electrophoresis of the genomic DNA extracted from the intestinal microflora of *Cynoglossus semilaevis* and *Cyprinus carpio* var*. Jian* using the CLU method. Lanes: *M* molecular marker. 1, *Cynoglossus semilaevis*; and 2, *Cyprinus carpio* var*. Jian*

On the agarose gel, a major high-intensity, discrete band of more than 10 kb was found for the CLU method. However, the smearing intensity of the degraded DNA was lower when the incubation temperatures were 60 °C for lysozyme and 30 °C for RNase than when incubation temperatures of 37 °C for lysozyme and 37 °C for RNase were used (Fig. 1). The ZBC method always generated an extended DNA band with a molecular weight of less than 10 kb and the highest intensity smearing signals in agarose gel electrophoresis. The QIA method generated a discrete band with a molecular weight similar to that obtained with the CLU method (Fig. 2). In addition, there was more obvious smearing of the degraded DNA in the QIA method than in the CLU method (Fig. 2). For the DNA samples extracted with the CLU method, a major high-intensity, discrete band with a high molecular weight of approximately 10 kb and limited smearing of degraded DNA were also observed (Fig. 3).

### Suitability of the DNA for PCR

**Fig. 4:**
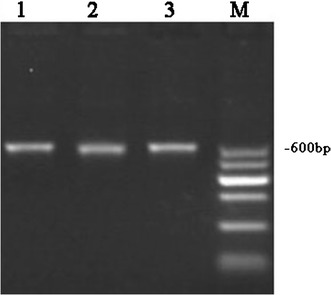
PCR products of the amplification of the V3-V4 variable region of the 16S rDNA using the DNA extracted with the CLU, ZBC and QIA methods as templates. Lanes: *M* molecular marker. 1, CLU method; 2, ZBC method; and 3, QIA method

The V3 variable region of the prokaryotic 16S rDNA was amplified, and an approximately 600 bp fragment was observed when the DNA samples extracted by the CLU, ZBC and QIA methods were used as templates (Fig. 4).

## Discussion

Several protocols for extracting DNA from the fish intestinal microflora, including physical and chemical methods, have been described. Generally, common physical disruption methods have been employed, such as freezing–thawing (Fan et al. 2014), sonication (Yang et al. 2006) and bead beating (Carrigg et al. 2008). In addition, a variety of chemical lysis approaches, including cetyl trimethyl ammonium bromide (Chapela et al. 2007) and Triton X-100 (Wang et al. 2012), have been used to obtain higher purity DNA. Many methods for DNA extraction employ lysozyme and proteinase K to quicken the process and increase the DNA yield. However, the incubation temperatures in the key steps are vital for the extraction process due to their influence on the activity of the enzymes used.

In the CLU method, the physical disruption and chemical lysis steps were combined, and the key parameters of the incubation temperatures of lysozyme and RNase were adjusted to 60 and 30 °C, respectively. The lysis of the cells is the first step of extracting DNA, and the lysis efficiency of any nucleic acid extraction procedure is critical in determining its success (Robe et al. 2003; Lipthay et al. 2004). It was reported that lysozyme had only a modest DNA extraction efficiency for Gram-positive bacteria and a few Gram-negative bacteria (Yu et al. 2013). To compensate for the low DNA extraction efficiency of lysozyme for most Gram-negative bacteria, a treatment step with proper ultrasonic disruption was used in the CLU method, which could break down the cell walls of Gram-negative bacteria and allowed good liberation of the DNA. In addition, the incubation temperature during the process of lysozyme lysis was usually maintained at 37 °C in previous studies (Kathiravan et al. 2015; Bag et al. 2016). However, some investigators noted that the activity of lysozyme from egg whites increased gradually with increasing temperature within the range of 25–65 °C (Liu et al. 2008). In this study, a lysozyme extract from egg whites was used. The disadvantage of the limited ability to extract DNA from gram-negative bacteria (Yu et al. 2013) was remedied by adjusting the incubation temperature to 60 °C and adding a treatment step with proper ultrasonic disruption. In addition, the temperature of 60 °C probably inactivated the DNase activity from the microbes and from the fish tissues, and the incubation temperatures of 30 °C for the RNase also partially inhibited DNase activity.

The quality of the extracted DNA was evaluated by the OD_260_/OD_280_ absorbance ratio (A_260_/A_280_). The DNA was considered to be sufficiently pure when the ratio was within the range of 1.7–2.1 (Wasko et al. 2003; Ferrara et al. 2006; Lopera-Barrero et al. 2008). An A_260_/A_280_ ratio lower than 1.8 suggested the presence of protein, salt or solvents, while an A_260_/A_280_ ratio above 2.0 usually indicated the presence of coextracted RNA. The finding of A_260_/A_280_ ratios above but close to 2.0 for most samples in this study indicated the presence of a small amount of RNA, most likely due to RNA coextraction with acidic phenol (the phenol chloroform had a pH of 5.2) (Tan and Yiap 2010). However, RNA in DNA extracts does not interfere with downstream applications (Wilfinger et al. 2006). Protein contamination would have caused low A_260_/A_280_ ratios (Wilfinger et al. 2006), which were not observed in the present study.

We further confirmed the quality of the isolated DNA by visualizing the samples on agarose gels containing the DNA-intercalating agent ethidium bromide. Although gel electrophoresis is not very sensitive for measuring the quality of DNA, it is a useful tool for analyzing stable RNA contamination and short-fragment DNA contamination, and it shows the average size of the isolated DNA. In this study, genomic DNA extracted with the CLU method formed a clear single band with a high molecular weight, which indicated DNA integrity. In addition, almost no smeared or fragmented DNA was observed in the lanes.

To confirm the validity of the CLU method, the other two fish, *C. semilaevis* and *C. carpio* var. *Jian*, were also used in the present study, and similar results were observed on the agarose gels. In addition, the ZBC and QIA methods were carried out for comparison. Genomic DNA extracted with the QIA method formed a clear single band with a high molecular weight, which indicated DNA integrity. However, the DNA extracted with the QIA method had more smearing than that extracted with the CLU method. The DNA band associated with the ZBC method was severely degraded and exhibited obvious smearing of highly fragmented DNA. This phenomenon was also seen consistently when the bead-beating method was applied to the cells (Krause et al. 2004; Villegas-Rivera et al. 2013).

For PCR-based community analysis, the quantity of DNA is not the key factor because trace DNA (> 500 ng) is adequate for PCR amplification and subsequent sequencing. In this study, the stained gel profile showed the PCR-amplified 16S rDNA obtained using the DNA samples extracted with the three methods as templates, and the results were consistent with the expected sizes of the PCR products. Additionally, nonspecific PCR amplification was not detected in any lane. This finding supported the suitability of the extracted DNA for molecular technologies, such as pyrosequencing, which employ short DNA fragments (Liu et al. 2007) but require clean and representative community DNA for in-depth analyses.

The CLU method is simple and cost effective, and it yields high-quality, unsheared, high molecular weight DNA, which is comparable to that obtained with commercially available kits. The extracted DNA has potential for use in critical molecular biology techniques, which are utilized as major tools to explore microbial communities. Further microbial diversity analyses based on the DNA extracted with the CLU method and comparative analyses with other methods are required.

## Abbreviations

CLU: combination of lysozyme and ultrasonic lysis
ZBC: Zirmil-beating cell disruption
QIA: QIAamp Fast DNA Stool Mini Kit
*C. carpio* var. *Koi*: koi carp *Cyprinus carpio* var. *Koi*
*C. carpio* var. *Jian*: Jian carp *Cyprinus carpio* var. *Jian*
*C. semilaevis*: *Cynoglossus semilaevis*
DNA: deoxyribonucleic acid
DNase: deoxyribonuclease
rRNA: rRibonucleic acid
16S rDNA: 16S ribosomal DNA
RNase: ribonuclease
PCR: polymerase chain reaction
ppt: parts per thousand
PBS: phosphate-buffered saline
SDS: sodium dodecyl sulfate
NaAc: sodium acetate
TE: tris-EDTA
Tris–HCl: tris (hydroxymethyl) aminomethane
EDTA: ethylenediaminetetraacetic acid
dNTP: deoxy-ribonucleoside triphosphate
MS-222: tricaine methanesulfonate
nm: nanometers
min: minutes
s: seconds
g: gravity
mL: milliliters
µL: microliters
mM: millimolar
M: molar
g: grams
mg: milligrams
µg: micrograms
ng: nanograms
kb: kilo base pairs
bp: base pairs
°C: degrees centigrade
OD: optical density
pH: hydrogen ion concentration
341F: 341 forward
805R: 805 reverse

## References

[CR1] Bag S, Saha B, Mehta O, Anbumani D, Kumar N, Dayal M, Pant A, Kumar P, Saxena S, Allin KH, Hansen T, Arumugam M, Vestergaard H, Pedersen O, Pereira V, Abraham P, Tripathi R, Wadhwa N, Bhatnagar S, Prakash VG, Radha V, Anjana RM, Mohan V, Takeda K, Kurakawa T, Nair GB, Dasa B (2016). An improved method for high quality metagenomics DNA extraction from human and environmental samples. Sci Rep.

[CR2] Brown K, DeCoffe D, Molcan E, Gibson DL (2012). Diet-induced dysbiosis of the intestinal microbiota and the effects on immunity and disease. Nutrients.

[CR3] Carrigg C, Rice O, Kavanagh S, Collins G, O’Flaherty V (2008). DNA extraction method affects microbial community profiles from soils and sediment. Appl Microbiol Biotechnol.

[CR4] Chapela MJ, Sotelo CG, Pérez-Martín RI, Pardo MÁ, Pérez-Villareal B, Gilardi P, Riesec J (2007). Comparison of DNA extraction methods from muscle of canned tuna for species identification. Food Control.

[CR5] Fan WG, Li XM, Yang LJ, Huo GC (2014). Comparison of DNA extraction methods for polymerase chain reaction-denaturing gradient gel electrophoresis (PCR-DGGE) analysis of the infant fecal microbial. Afr J Microbiol Res.

[CR6] Ferrara GB, Murgia B, Parodi AM, Valisano L, Cerrano C, Palmisano G, Bavestrello G, Sara M (2006). The assessment of DNA from marine organisms via a modified salting-out protocol. Cell Mol Biol Lett.

[CR7] Gajardo K, Rodiles A, Kortner TM, Krogdahl Å, Bakke AM, Merrifield DL, Sørum H (2016). A high-resolution map of the gut microbiota in Atlantic salmon (*Salmo salar*): a basis for comparative gut microbial research. Sci Rep.

[CR8] Ganguly S, Prasad A (2011). Microflora in fish digestive tract plays significant role in digestion and metabolism. Rev Fish Biol Fish.

[CR9] Gatesoupe FJ (1999). The use of probiotics in aquaculture. Aquaculture.

[CR10] Hoffmann F, Rapp HT, Reitner J (2006). Monitoring microbial community composition by fluorescence in situ hybridization during cultivation of the marine cold-water sponge *Geodia barretti*. Mar Biotechnol.

[CR11] Hu X, Xu B, Yang Y, Liu D, Yang M, Ji W, Shen H, Zhou X, Ma X (2013). A high throughput multiplex PCR assay for simultaneous detection of seven aminoglycoside-resistance genes in Enterobacteriaceae. BMC Microbiol.

[CR12] Kathiravan MN, Gim GH, Ryu J, Kim PI, Lee CW, Kim SW (2015). Enhanced method for microbial community DNA extraction and purification from agricultural yellow loess soil. J Microbiol.

[CR13] Krause DO, Smith WJ, McSweeny CS (2004). Use of community genome arrays (CGAs) to assess the effects of *Acacia angustissima* on rumen ecology. Microbiol.

[CR14] Li XM, Yu YH, Xie SQ, Yan QY, Chen YH, Dong XL (2011). PCR-DGGE fingerprinting analysis on intestinal microbial community of three indoor rearing fishes. Acta Hydrobiol Sin.

[CR15] Li D, Chen B, Zhang L, Gaur U, Ma T, Jie H, Zhao G, Wu N, Xu Z, Xu H, Yao Y, Lian T, Fan X, Yang D, Yang M, Zhu Q, Trask JS (2016). The musk chemical composition and microbiota of Chinese forest musk deer males. Sci Rep.

[CR16] Lipthay JRD, Enzinger C, Johnsen K, Aamand J, Sørensen SJ (2004). Impact of DNA extraction method on bacterial community composition measured by denaturing gradient gel electrophoresis. Soil Biol Biochem.

[CR17] Liu Z, Lozupone C, Hamady M, Bushman FD, Knight R (2007). Short pyrosequencing reads suffice for accurate microbial community analysis. Nucleic Acids Res.

[CR18] Liu H, Wang FS, Chu J (2008). Study on some enzymological properties and activity influencing factors of egg white lysozyme. Chin J Biochem Pharm.

[CR19] Lopera-Barrero NM, Povh JA, Ribeiro RP, Gomes PC, Jacometo CB, Silva Lopes TS (2008). Comparison of DNA extraction protocols of fish fin and larvae samples: modified salt (NaCl) extraction. Cien Inv Agr.

[CR20] Morgan XC, Tickle TL, Sokol H, Gevers D, Devaney KL, Ward DV, Reyes JA, Shah SA, LeLeiko N, Snapper SB, Bousvaros A, Korzenik J, Sands BE, Xavier RJ, Huttenhower C (2012). Dysfunction of the intestinal microbiome in inflammatory bowel disease and treatment. Genome Biol.

[CR21] Narrowe AB, Albuthilantz M, Smith EP, Bower KJ, Roane TM, Vajda AM, Miller CS (2015). Perturbation and restoration of the fathead minnow gut microbiome after low-level triclosan exposure. Microbiome.

[CR22] Robe P, Nalin R, Capellano C, Vogel TM, Simonet P (2003). Extraction of DNA from soil. Eur J Soil Biol.

[CR23] Sambrook J, Fritsch EF, Maniatis T (1989). Molecular cloning: a laboratory manual.

[CR24] Stearns JC, Lynch MD, Senadheera DB, Tenenbaum HC, Goldberg MB, Cvitkovitch DG, Croitoru K, Moreno-Hagelsieb G, Neufeld JD (2011). Bacterial biogeography of the human digestive tract. Sci Rep.

[CR25] Sugita H, Kawasahi J, Deguchi Y (1997). Production of amylase by the intestinal microflora in cultured freshwater fish. Lett Appl Microbiol.

[CR26] Tan SC, Yiap BC (2010). DNA, RNA, and protein extraction: the past and the present. J Biomed Biotechnol.

[CR27] Villegas-Rivera G, Vargas-Cabrera Y, González-Silva N, Aguilera-García F, Gutiérrez-Vázquez E, Bravo-Patiño A, Cajero-Juárez M, Baizabal-Aguirre VM, Valdez-Alarcón JJ (2013). Evaluation of DNA extraction methods of rumen microbial populations. World J Microbiol Biotechnol.

[CR28] Wang XF, Du ZW, Wu M, Zhang YC, Jiang Y, Zhang GZ (2012). DNA extraction from formalin-fixed and paraffin-embedded tissues by triton X-100 for effective amplification of EGFR gene by polymerase chain reaction. Chem Res Chinses U.

[CR29] Wasko AP, Martins C, Oliveira C, Foresti F (2003). Non-destructive genetic sampling in fish. An improved method for DNA extraction from fish fins and scales. Hereditas.

[CR30] Wilfinger WW, Mackey K, Chomczynski P (2006). Assessing the quantity, purity and integrity of RNA and DNA following nucleic acid purification.

[CR31] Xia JH, Lin G, Fu GH, Wan ZY, Lee M, Wang L, Liu XJ, Yue GH (2014). The intestinal microbiome of fish under starvation. Bmc Genom.

[CR32] Yang ZH, Xiao Y, Zeng GM, Liu YG, Deng JH (2006). DNA extraction methods of compost for molecular ecology analysis. Environ Sci.

[CR33] Yu LP, Sun BG, Li J, Sun L (2013). Characterization of a c-type lysozyme of *Scophthalmus maximus*: expression, activity, and antibacterial effect. Fish Shellfish Immunol.

[CR34] Zoetendal EG, Heilig HG, Klaassens ES, Booijink CC, Kleerebezem M, Smidt H, de Vos WM (2006). Isolation of DNA from bacterial samples of the human gastrointestinal tract. J Nat Protoc.

